# Loneliness and problematic use of conversational artificial intelligence among college students: the serial mediating roles of social anxiety and self-control

**DOI:** 10.3389/fpsyg.2026.1950646

**Published:** 2026-09-30

**Authors:** Bingzhi Li, Lu Shi, Xiaofei Bu, Hanwei Zhou

**Affiliations:** 1 School of Marxism, Xinjiang Normal University, Urumqi, China; 2 School of Political Science and Law, Xinjiang Normal University, Urumqi, China

**Keywords:** college students, loneliness, problematic use of conversational artificial intelligence, self-control, social anxiety

## Abstract

**Objectives:**

Loneliness is an important public health concern associated with individuals’ physical and mental wellbeing. As conversational artificial intelligence (CAI) becomes more widely used, concerns about its problematic use have attracted increasing attention. However, the link between loneliness and problematic use of conversational artificial intelligence (PUCAI) remains insufficiently understood. The current research explored the link between loneliness and PUCAI among college students, with social anxiety and self-control evaluated as serial mediators. These findings provide preliminary empirical insight into the psychological correlates of PUCAI and may inform future research and educational efforts aimed at promoting healthy and responsible CAI use.

**Methods:**

Using convenience sampling, this cross-sectional study analyzed data from 400 undergraduate students enrolled at a university in western China. Research instruments included the Loneliness Scale, the PUCAI Scale, the Social Anxiety Scale, and the Self-Control Scale. Descriptive statistics and Pearson correlations were computed with IBM SPSS Statistics 26.0. Serial mediation was examined with Hayes’ PROCESS macro (Model 6), and indirect effects were evaluated using 5,000 bootstrap resamples with 95% confidence intervals.

**Results:**

Greater loneliness was linked to higher PUCAI. Social anxiety and self-control each showed significant indirect associations, and the proposed statistical model indicated a significant serial indirect effect involving these two variables.

**Conclusion:**

The results offer further insight into the potential psychological pathways associated with PUCAI among college students. The findings suggest that loneliness, social anxiety, and self-regulatory capacities may represent potentially relevant factors to consider in future research and prevention efforts aimed at promoting healthy and responsible CAI use.

## 1 Introduction

With the advancement of digital technology, people are engaging more frequently with conversational agents capable of human-like communication (Khatri et al., 2018). Such technologies are commonly referred to as conversational artificial intelligence (CAI). CAI denotes programs developed using AI and natural language processing that can with users in a natural and intelligent manner across multiple modalities (Henkel et al., 2020). Common examples include chatbots and smart assistants (Guzman and Lewis, 2020). In recent years, with continuous advances in AI technologies, the human-computer interaction experience has become increasingly natural, driving the widespread application of CAI in learning, work and everyday life (Montenegro et al., 2019). Furthermore, the rapid proliferation of CAI is profoundly transforming the way college students engage digitally. For college students, CAI can be used not only to assist with academic tasks, but also to provide emotional support. However, its personalized responses and human-like interactions may also pose potential risks. Some students may be exposed to inappropriate content while using these systems, or may gradually develop an emotional dependence on CAI (Ramadan, 2021). Existing research indicates that as the frequency of user interaction with CAI increases, individuals may form stronger psychological bonds with it, thereby increasing the risk of problematic use of CAI (PUCAI) (Heng and Zhang, 2025). At the same time, loneliness is a negative emotional experience closely linked to social cognitive processing and is relatively common among college students (Zhao and Kou, 2024). Existing evidence suggests a certain link between loneliness and PUCAI (Jain et al., 2026). However, underlying psychological mechanisms through which loneliness influences PUCAI among college students remain to be further elucidated. Consequently, this research aims to investigate the association between loneliness and PUCAI, thereby providing a theoretical basis for universities to develop targeted guidelines for responsible CAI use and mental health intervention.

### 1.1 Loneliness and PUCAI

PUCAI denotes excessive, compulsive, or inadequately controlled engagement with CAI that results in negative consequences for individuals’ academic functioning, daily life, interpersonal relationships, or mental health (Pentina et al., 2023; Sun, 2023). CAI is capable of social interaction with users, whether verbally or in writing, and provides personalized, immediate and highly human-like responses (Chaves and Gerosa, 2021). Furthermore, some CAI systems can continuously adjust their interaction style based on user input, exhibiting certain characteristics of adaptability, autonomy and persistent memory. These features may render them more appealing than other intelligent agents in terms of the interactive experience, and may in particular lead some users to develop PUCAI (Hinduja and Patchin, 2026). Loneliness refers to a persistent negative subjective emotional experience arising in situations where people perceive social relationships as falling short of their expectations (Cacioppo et al., 2010). Loneliness among college students has attracted growing research interest (Käll et al., 2021). Existing research indicates that there is an association between loneliness and PUCAI (Uğuz et al., 2026). Prior evidence has linked greater loneliness to problematic social media and Internet use, suggesting that loneliness might represent a person-related vulnerability associated with problematic technology use (Dey and Roy, 2026; Jing et al., 2025). It can therefore be inferred that loneliness may increase the risk of PUCAI. Accordingly, the following hypothesis is formulated:

H1: Loneliness positively predicts PUCAI among college students.

### 1.2 The mediating role of social anxiety

Social anxiety encompasses negative emotional, cognitive, and behavioral responses that occur in interpersonal interactions or social contexts, typically involving concerns about negative evaluation, nervousness during social interactions, and fear of social failure or rejection (Rapee and Heimberg, 1997). Existing research indicates that loneliness is linked to social anxiety among college students (Cavanaugh and Buehler, 2016). Individuals experiencing greater loneliness tend to report elevated social anxiety. Evidence from longitudinal studies further indicates that loneliness at baseline predicts subsequent levels of social anxiety (Lim et al., 2016). Social anxiety may then increase the risk of PUCAI. Individuals with elevated social anxiety often experience heightened concerns about negative evaluation and rejection during face-to-face social encounters and may be more likely to avoid direct interpersonal interaction (Huan et al., 2014). However, CAI, characterized by immediate responses, low social pressure, a high degree of control and human-like interactions, can provide individuals with social anxiety with a relatively safe interactive environment (Fox and Gambino, 2021). Existing evidence has documented an association between social anxiety and PUCAI (Merrill et al., 2025). Together with evidence linking loneliness to social anxiety, these associations indicate that social anxiety might act as a potential indirect pathway linking loneliness to PUCAI. Accordingly, the following hypothesis is formulated:

H2: Social anxiety mediates the relationship between loneliness and PUCAI among college students.

### 1.3 The mediating role of self-control

Self-control denotes the capacity to overcome distractions and inhibit impulses in daily life, and to work toward desired outcomes through the regulation of affect, cognition, and behavior (Baumeister et al., 2007). Prior findings indicate that loneliness is linked to self-control (Yue et al., 2022). Loneliness may arise when individuals perceive their social relationships as falling short of their expectations and is often accompanied by distress, loss, and feelings of exclusion (He and Wang, 2025). When individuals experience loneliness, they often need to devote more cognitive and emotional regulatory resources to cope with the psychological stress caused by a lack of social connection, thereby reducing the self-regulatory resources available for impulse control, delayed gratification, and maintaining goal-directed behavior (Gan and Wang, 2026). Consequently, loneliness may weaken an individual’s self-control. Lower self-control may further increase the risk of PUCAI. Previous research has demonstrated a link between reduced self-control and problematic technology use (Shen et al., 2026). Compared to traditional digital technologies, CAI features instant feedback, low cost of access, continuous availability, sustained interaction, and human-like responses, which may further enhance its appeal to individuals with reduced self-control and thus increase their susceptibility to PUCAI. Accordingly, the following hypothesis is formulated:

H3: Self-control mediates the relationship between loneliness and PUCAI among college students.

### 1.4 The chain intermediary role of social anxiety and self-control

Prior evidence has demonstrated a link between social anxiety and self-control (Li et al., 2022). This relationship may be understood in light of ego-depletion theory, which holds that self-control relies on finite regulatory resources (Leith and Baumeister, 1996). When individuals experience social anxiety, it is commonly associated with tension, worry, heightened concerns about negative evaluation, and social avoidance. For coping with these emotional reactions, individuals must continuously invest cognitive and emotional regulatory resources, which may interfere with the regulatory capacity required for impulse inhibition, goal maintenance, and behavioral regulation, potentially weakening effective self-control (Wang et al., 2025). Therefore, social anxiety may constitute an important affective factor associated with reduced self-control. The I-PACE model proposed by Brand et al. aims to explain the development and maintenance of problematic use of specific Internet applications by considering the interactions among person-related characteristics, affective and cognitive responses, and executive-control processes. The person component refers to relatively stable individual predispositions, including psychopathological characteristics, personality traits, and social cognitions. In response to internal or external situational triggers, these predispositions may be associated with specific affective and cognitive responses, such as coping styles, Internet-related cognitive biases, attentional processes, cue-reactivity, and craving. These affective and cognitive responses may, in turn, interact with executive-control processes, including inhibitory control and decision-making related to the use of specific Internet applications (Brand et al., 2016). Guided by the I-PACE framework, the present study conceptualized loneliness as a person-related vulnerability factor, social anxiety as an affective response, and self-control as an executive-control-related factor. College students experiencing greater loneliness may report greater interpersonal concerns and social anxiety when they perceive their social connectedness as insufficient. From a self-regulation perspective, managing persistent social anxiety and concerns about negative evaluation may place demands on regulatory resources, providing a theoretical rationale for positioning self-control after social anxiety in the proposed statistical model. Lower self-control may, in turn, be associated with greater difficulty regulating engagement with the immediate and compensatory rewards offered by CAI and with higher levels of PUCAI. On this theoretical basis, the present study specified a serial indirect model in which social anxiety precedes self-control in the statistical ordering between loneliness and PUCAI. This ordering reflects a theoretically motivated model specification rather than an established temporal or causal sequence, and alternative directional or reciprocal relationships among these variables remain possible. Accordingly, the following hypothesis is formulated:

H4: Social anxiety and self-control sequentially mediate the association between loneliness and PUCAI among college students.

## 2 Materials and methods

### 2.1 Participants

Using convenience sampling, undergraduate students from a university in western China were recruited to complete an online questionnaire. The survey participants included students from freshman through senior year. The online questionnaire was administered through Wenjuanxing, and the researcher invited some course instructors to assist in recruiting participants by sharing the questionnaire link or QR code with students to collect data. Participation was informed, voluntary, and anonymous. Before formally answering the questionnaire, participants were asked to review the informed consent information and indicate their consent to participate. The informed consent form detailed the research objectives, confidentiality protections, the voluntary nature of participation, and participants’ right to withdraw at any point without penalty. A total of 421 completed questionnaires were obtained. After data collection, invalid questionnaires were identified and excluded based on the following criteria: (a) incomplete responses (i.e., items left blank); (b) careless responses with obvious logical inconsistencies (e.g., contradictory answers to related questions); (c) failure to pass attention-check questions randomly inserted by the Wenjuanxing system; and (d) abnormally short or long response times (a reasonable response time was approximately 3–5 s per question). Following data screening, 21 invalid responses were removed, leaving a final analytic sample of 400 and a valid response rate of 95.0%. Participants had a mean age of 20.36 years (SD = 1.73), with 280 identifying as male (70.0%) and 120 as female (30.0%). First-, second-, third-, and fourth-year students accounted for 18.25, 18.25, 27.25, and 36.25%, respectively.

### 2.2 Measures

#### 2.2.1 Loneliness scale

Loneliness was measured using the UCLA Loneliness Scale (ULS-8) (Hays and DiMatteo, 1987). The ULS-8 comprises eight items rated on a four-point Likert scale ranging from 1 (never) to 4 (often). Six items were worded in a negative/lonely direction and two items were worded in a positive/non-lonely direction. Total scores range between 8 and 32, with higher scores reflecting greater loneliness. The Chinese version has shown satisfactory psychometric properties (Shi et al., 2023). In this study, Cronbach’s *α* was 0.81.

#### 2.2.2 PUCAI scale

PUCAI was assessed using the six-item measure reported by Hu et al. (2023). This measure was derived from the Bergen Social Media Addiction Scale, which was itself adapted from the Bergen Facebook Addiction Scale by replacing references to ‘Facebook’ with ‘social media’ (Andreassen et al., 2017; Andreassen et al., 2012). Hu et al. subsequently adapted the six BSMAS items to the CAI context by replacing ‘social media’ with ‘CAI’. In the present study, the six items reported by Hu et al. were adopted without further modification to their substantive content. An example item is ‘I spend a lot of time thinking about CAI’. Items were rated on a 5-point Likert scale ranging from 1 (very rarely) to 5 (very often). Total scores range between 6 and 30, with higher scores reflecting greater PUCAI. In this study, Cronbach’s *α* was 0.82.

#### 2.2.3 Social anxiety scale

Social anxiety was measured using the Chinese adaptation of the Interaction Anxiousness Scale (IAS) developed by Leary (1983) and Peng et al. (2004). The scale consists of 15 items, including “I feel nervous when I need to talk to a teacher or supervisor.” Participants rated each item on a 5-point Likert scale ranging from 1 (strongly disagree) to 5 (strongly agree). Items 3, 6, 10, and 15 were reverse-scored, and total scores range between 15 and 75, with higher scores reflecting higher levels of social anxiety. In this study, Cronbach’s *α* was 0.86.

#### 2.2.4 Self-control scale

Self-control was assessed using the Chinese Self-Control Scale (SCS) revised by Tan and Guo (2008). The scale consists of 19 items across five dimensions: impulse control, healthy habits, resistance to temptation, focus on work or study, and moderation in leisure activities. Items were rated on a 5-point Likert scale ranging from 1 (strongly disagree) to 5 (strongly agree). Items 2–4, 6–10, 12, 13, and 15–19 were reverse-scored, and total scores range between 19 and 95, with higher scores reflecting greater self-control. In this study, Cronbach’s *α* was 0.86.

### 2.3 Data analysis

All analyses were performed in IBM SPSS Statistics 26.0. Descriptive statistics and Pearson correlations were calculated, followed by assessments of common method bias using Harman’s single-factor procedure and multicollinearity using VIFs. All VIFs were below 10, suggesting no serious multicollinearity. Before the mediation analysis, all continuous variables were standardized into Z scores so that the estimated associations could be expressed in standard-deviation units and the magnitudes of different path coefficients could be compared. The proposed serial mediation model was estimated using Hayes’s PROCESS Model 6 (Hayes, 2013), with loneliness as the predictor, social anxiety and self-control as the first and second mediators, respectively, and PUCAI as the outcome. Indirect effects were examined through 5,000 bootstrap resamples and 95% bias-corrected confidence intervals, with intervals excluding zero indicating statistical significance. Because all focal variables were measured at a single time point, the estimated indirect effects should be interpreted as statistical associations consistent with the hypothesized model rather than as evidence of temporal ordering or causal mechanisms. Standardized regression coefficients (*β*), based on Z-standardized continuous variables, are reported in Table 1 and Figure 1. In contrast, Table 2 reports unstandardized direct and indirect effect estimates (*b*) obtained from PROCESS Model 6 using variables in their original metric. Both analyses were based on the same analytic sample and included gender and academic year as covariates. All tests were two-tailed at *α* = 0.05.

**Table 1:** Regression results of the mediation model.

Regression equation		Overall fit index			Significance of regression coefficient	
Result variable	Predictive variable	*R*	*R^2^*	*F*	*β*	*t*
PUCAI	Gender	0.422	0.178	28.666	−0.099	−1.978*
	Grade				0.036	0.721
	Loneliness				0.410	8.991***
Social anxiety	Gender	0.332	0.110	16.355***	−0.041	−0.937
	Grade				−0.023	−0.487
	Loneliness				0.327	6.902***
Self-control	Gender	0.433	0.187	22.760***	−0.004	−0.091
	Grade				0.026	0.571
	Loneliness				−0.154	−3.214**
	Social anxiety				−0.356	−7.397***
PUCAI	Gender	0.492	0.242	25.175***	−0.085	−1.928
	Grade				0.045	1.018
	Loneliness				0.324	6.882***
	Social anxiety				0.074	1.483
	Self-control				−0.228	−4.694***

*p < 0.05, **p < 0.01, ***p < 0.001.

**Figure 1:**
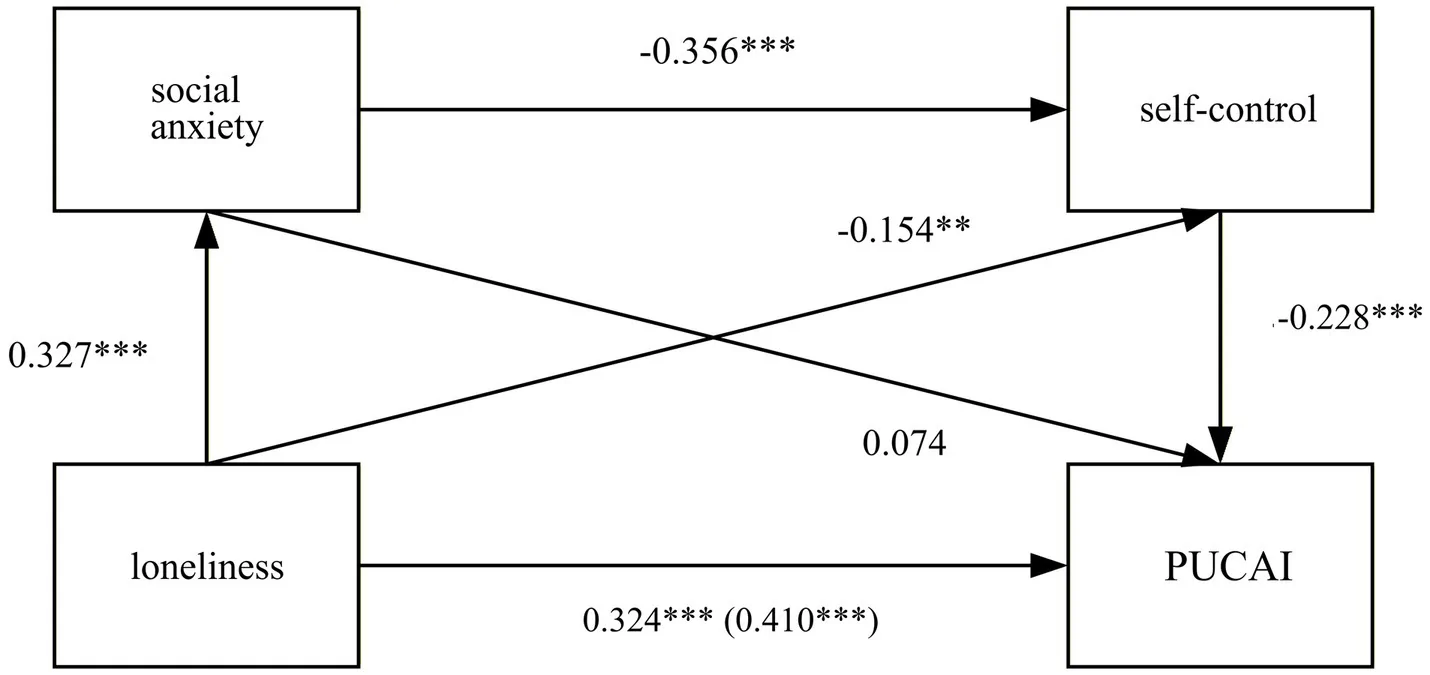
XXX.

**Table 2:** Bootstrap tests of the direct and indirect effects.

Path	Effect	Boot SE	95% bootstrap CI	Proportion of total effect (%)
Total effect	0.422	0.047	[0.330, 0.515]	100.00%
Direct effect	0.334	0.049	[0.239, 0.429]	79.15%
Total indirect effect	0.088	0.022	[0.048, 0.134]	20.85%
Path 1: Loneliness→social anxiety→PUCAI	0.025	0.018	[0.010, 0.061]	5.92%
Path 2: Loneliness→self-control→PUCAI	0.036	0.014	[0.015, 0.070]	8.53%
Path 3: Loneliness→social anxiety→self-control→PUCAI	0.027	0.008	[0.015, 0.047]	6.40%

## 3 Results

### 3.1 Common method bias test

Harman’s single-factor procedure was conducted as a preliminary assessment of potential common method bias. The unrotated exploratory factor analysis extracted 16 factors with eigenvalues above 1, and the largest factor accounted for 12.86% of the total variance. As this proportion was below the conventional 40% threshold, common method bias was unlikely to represent a serious concern. However, given the limitations of Harman’s single-factor test, the possibility of common method bias cannot be completely ruled out.

### 3.2 Description and correlation

As shown in Table 3, loneliness was positively correlated with social anxiety (*r* = 0.328, *p* < 0.01) and PUCAI (*r* = 0.411, *p* < 0.01), whereas it was negatively correlated with self-control (*r* = −0.271, *p* < 0.01). Social anxiety was positively related to PUCAI (*r* = 0.276, *p* < 0.01) but negatively related to self-control (*r* = −0.407, *p* < 0.01). A negative correlation was also observed between self-control and PUCAI (*r* = −0.346, *p* < 0.01). All correlations were statistically significant and in the hypothesized directions, providing preliminary support for the proposed mediation model.

**Table 3:** Descriptive statistics and correlations among the study variables (*n* = 400).

Variables	M	SD	1	2	3	4
Loneliness	19.995	3.966	1			
Social anxiety	38.475	8.071	0.328**	1		
Self-control	56.423	11.742	−0.271**	−0.407**	1	
PUCAI	18.198	4.091	0.411**	0.276**	−0.346**	1

**p < 0.01.

### 3.3 Analysis of the mediating effect

PROCESS Model 6 was used to examine the proposed serial mediation model while controlling for gender and academic year. The regression results presented in Table 1 showed that loneliness was positively associated with PUCAI (*β* = 0.410, *p* < 0.001) and social anxiety (*β* = 0.327, *p* < 0.001), but negatively associated with self-control (*β* = −0.154, *p* < 0.01). Social anxiety, in turn, was inversely related to self-control (*β* = −0.356, *p* < 0.001). In the final model, self-control showed a significant negative association with PUCAI (*β* = −0.228, *p* < 0.001), whereas the social anxiety–PUCAI association was nonsignificant (*β* = 0.074, *p* > 0.05). The direct relationship between loneliness and PUCAI remained significant after accounting for both mediators (*β* = 0.324, *p* < 0.001). The tested mediation paths are presented in Figure 1.

Table 2 presents unstandardized total, direct, and indirect effect estimates (*b*) from PROCESS Model 6, whereas Table 1 and Figure 1 report standardized regression coefficients (*β*) based on Z-standardized continuous variables. The total effect of loneliness on PUCAI was 0.422, 95% CI [0.330, 0.515], of which 79.15% was attributable to the direct effect (0.334, 95% CI [0.239, 0.429]) and 20.85% to the combined indirect effect (0.088, 95% CI [0.048, 0.134]). The specific indirect effects were 0.025 through social anxiety (5.92, 95% CI [0.010, 0.061]), 0.036 through self-control (8.53, 95% CI [0.015, 0.070]), and 0.027 through the serial pathway involving social anxiety followed by self-control (6.40, 95% CI [0.015, 0.047]). Because none of these intervals included zero, all tested effects were statistically significant. These results supported significant indirect effects involving social anxiety and self-control, both independently and through a statistically specified serial indirect effect. Given the cross-sectional design, these findings should be interpreted as statistical associations consistent with the proposed model rather than as evidence of temporal ordering or a causal psychological mechanism.

## 4 Discussion

### 4.1 Direct effect of loneliness on PUCAI

The findings revealed a significant positive relationship between loneliness and PUCAI among college students, providing support for Hypothesis 1 and aligning with previous empirical evidence (Lai et al., 2025; Ramazan et al., 2023). The compensatory Internet use framework proposes that individuals experiencing loneliness and psychological distress may engage with Internet-based technologies to cope with real-world pressures or address unmet needs for social connections and emotional support within their offline interpersonal relationships (Kardefelt-Winther, 2014). Based on this theory, due to perceived deficiencies in their offline social relationships, college students with higher levels of loneliness often have a greater desire for companionship, responsiveness, and emotional support. CAI can provide immediate and controllable responses, reduce the perceived risk of negative interpersonal evaluation, and simulate human-like interaction (Skjuve et al., 2021), which can, to a certain extent, satisfy their needs for emotional compensation and social substitution. Furthermore, previous studies have suggested that individuals reporting greater loneliness tend to engage with CAI in search of companionship and relief from loneliness, thereby significantly increasing their intentions to use CAI (Peng et al., 2025). Although an increased motivation to use CAI does not necessarily constitute problematic use, repeated reliance on CAI for emotional compensation may reinforce usage behavior. Such a pattern of reliance may be associated with more frequent, dependent, or poorly controlled CAI use, which may represent a potential risk factor for PUCAI. Therefore, loneliness may constitute a relevant person-related vulnerability linked to PUCAI among college students.

### 4.2 Mediating effect of social anxiety

Results from the present study revealed that the association between loneliness and PUCAI was partially explained by social anxiety among college students, supporting Hypothesis 2 and being in line with earlier research (Cokluk, 2026). Existing evidence suggests that loneliness has been linked to social anxiety (Huan et al., 2014). Based on the evolutionary framework of loneliness, loneliness functions as a signal of insufficient social connectedness and motivates individuals to restore social bonds. However, when loneliness persists, it may increase sensitivity to rejection-related cues and foster threat-oriented interpretations of interpersonal information (Cacioppo and Hawkley, 2009). These cognitive biases may increase sensitivity to rejection, exclusion, and negative evaluation, thereby contributing to greater social anxiety and avoidance tendencies (Rapee and Heimberg, 1997). Social anxiety may be associated with greater levels of PUCAI (Almaiah et al., 2022). Individuals with greater social anxiety usually show heightened concerns about being negatively evaluated or rejected in face-to-face settings, which may reduce their willingness to engage actively in offline social interactions (High and Caplan, 2009). In contrast, CAI can simulate relatively natural interpersonal interactions and provide individuals with social anxiety a relatively safe and relaxed virtual interaction environment (Nomura et al., 2020). It may therefore serve as an alternative source of companionship, belonging, and emotional support for socially anxious individuals (Skjuve et al., 2021). However, repeated reliance on CAI to alleviate interpersonal anxiety or compensate for unmet social needs may reinforce its use and gradually develop into dependent, repetitive, or poorly controlled usage patterns, thereby further increasing the likelihood of PUCAI (Drouin et al., 2022). Taken as a whole, the available evidence suggests that social anxiety was identified as a statistical indirect factor associated with the relationship between loneliness and PUCAI.

### 4.3 Mediating effect of self-control

Results from the present study indicated that self-control partially explained the relationship between loneliness and PUCAI among college students, supporting Hypothesis 3 and extending prior research (Heidari et al., 2025). Greater loneliness may be accompanied by perceived deficiencies in people’s social connectedness and social support (Guo et al., 2026). Persistent loneliness and the regulation of its associated psychological distress may place sustained demands on self-regulatory capacities, potentially undermining individuals’ capacity to sustain effective self-regulation and behavioral control when faced with stress, temptation, or situations offering instant gratification (Li et al., 2021). Consequently, loneliness may be linked to lower self-control. Lower self-control was associated with higher PUCAI, although the directionality of this association requires further investigation (Kara, 2026). I-PACE framework conceptualizes self-control as the broad capacity for self-regulation that is closely related to executive control, including the ability to inhibit impulsive behavior, manage usage goals, and persevere toward long-term goals (Brand et al., 2019). College students reporting lower self-control may have greater difficulty regulating their engagement with CAI, particularly because such technologies provide immediate feedback, easy access, and convenient assistance. They may therefore tend to rely on CAI to escape academic stress or satisfy unmet social needs. When this pattern of use becomes repetitive, dependent, or poorly controlled, the risk of PUCAI may increase. Overall, the available evidence indicates that self-control may represent a potential indirect pathway linking loneliness to PUCAI.

### 4.4 Chain mediating effect of social anxiety and self-control

Results from the present study indicated that the association between loneliness and PUCAI was statistically associated with an indirect effect involving social anxiety and self-control, supporting Hypothesis 4. This finding is broadly consistent with prior research showing associations between social anxiety and self-control and is compatible with the theoretically specified model (Ma et al., 2025). From the perspective of the I-PACE framework, loneliness may be conceptualized as a person-related vulnerability factor, social anxiety as an affective response, and self-control as an executive-control-related factor. This conceptualization provides a theoretical rationale for specifying social anxiety before self-control in the present model. Specifically, college students with higher levels of loneliness may perceive their social connectedness as insufficient and consequently experience greater tension, interpersonal apprehension, and concerns about negative evaluation during social interactions, thereby reporting greater social anxiety (Reinwarth et al., 2024). Persistent social anxiety may place sustained demands on cognitive and emotional regulation, thereby interfering with the effective deployment of self-control (Nigg, 2017). Lower self-control may be associated with greater difficulty in regulating CAI engagement and maintaining reasonable usage boundaries, which may be linked to higher levels of PUCAI. These findings suggest that PUCAI among college students may be associated not only with the technological convenience of CAI but also with users’ emotional experiences, perceived social connectedness, and self-regulatory capacities. Importantly, however, the I-PACE framework emphasizes dynamic interactions among person-related predispositions, affective and cognitive responses, and executive-control processes rather than a fixed unidirectional sequence. Accordingly, the present findings should be interpreted as supporting one theoretically motivated statistical model rather than demonstrating a temporal or causal process from loneliness to social anxiety, self-control, and PUCAI. Alternative directional or reciprocal relationships among these variables remain plausible and should be examined in future longitudinal or experimental studies.

## 5 Limitations and future research

Despite these findings, several methodological limitations should be acknowledged. First, the use of cross-sectional data prevents determination of the temporal precedence or causal direction of associations among loneliness, social anxiety, self-control, and PUCAI. Although the analysis examined a theoretically derived serial mediation model, alternative directional and reciprocal associations remain plausible. Future studies could implement longitudinal, cross-lagged panel, or experimental approaches to examine how these associations among the study variables unfold over time and to strengthen causal inference. Second, participants were recruited through convenience sampling from one institution, potentially constraining the broader applicability of the findings. Students across different regions, academic majors and sociocultural contexts may report different levels of loneliness and patterns of CAI use. Future research could draw on more heterogeneous samples from different universities and regions and examine whether the proposed model is invariant across demographic and educational groups. Third, the use of self-report measures might introduce shared method effects. Although no dominant single factor emerged from Harman’s test, the limitations of this procedure mean that common method effects cannot be completely excluded. Future studies could adopt more robust methodological approaches and integrate multiple methods and data sources. Finally, the current research examined only sequential mediating effects of social anxiety and self-control. Other psychological and situational mechanisms, such as rumination and academic stress, may also explain the link between loneliness and PUCAI. Subsequent research could examine these variables as potential mediators or moderators to deepen understanding of the psychological processes linking loneliness to PUCAI.

## 6 Conclusions and implications

Three principal findings emerged from this study. First, loneliness showed an association with PUCAI among college students. Second, social anxiety and self-control were involved in a significant serial indirect effect in the association between loneliness and PUCAI. Third, social anxiety and self-control constituted a sequential indirect pathway linking loneliness to PUCAI. The results offer implications for both theory and practice. Theoretically, they provide preliminary support for social anxiety and self-control as potential psychological pathways linking loneliness to PUCAI and extend research on problematic technology use to the context of CAI. From a practical perspective, the findings highlight loneliness, social anxiety, and self-regulatory capacities as potentially relevant factors that universities and educators may consider when developing AI literacy initiatives to promote healthy and responsible CAI use. In addition, universities and educators may also pay attention to students’ loneliness, social anxiety, and self-control abilities. Specifically, through group-based counseling, enhanced social support, interpersonal skills development, and self-management training, educators may help college students alleviate loneliness and social anxiety while strengthening their self-control abilities, thereby promoting their psychological wellbeing. However, the present cross-sectional findings do not establish that modifying loneliness, social anxiety, or self-control would lead to changes in PUCAI, and the effectiveness of such approaches in reducing PUCAI should be evaluated in future longitudinal or intervention studies.

## Data Availability

The raw data supporting the conclusions of this article will be made available by the authors, without undue reservation.
